# Urinary Extracellular Vesicles Are a Novel Tool to Monitor Allograft Function in Kidney Transplantation: A Systematic Review

**DOI:** 10.3390/ijms221910499

**Published:** 2021-09-28

**Authors:** Liang Wu, Karin Boer, Wouter W. Woud, Suwasin Udomkarnjananun, Dennis A. Hesselink, Carla C. Baan

**Affiliations:** 1 Erasmus MC Transplant Institute, Department of Internal Medicine, University Medical Center Rotterdam Erasmus MC, Doctor Molewaterplein 40, 3015 GD Rotterdam, The Netherlands; karin.boer@erasmusmc.nl (K.B.); w.woud@erasmusmc.nl (W.W.W.); s.udomkarnjananun@erasmusmc.nl (S.U.); d.a.hesselink@erasmusmc.nl (D.A.H.); c.c.baan@erasmusmc.nl (C.C.B.); 2 Department of Nephrology, The First Affiliated Hospital of Shaoyang University, Shaoyang 422000, China; 3 Division of Nephrology, Department of Medicine, Faculty of Medicine, Chulalongkorn University and King Chulalongkorn Memorial Hospital, 1873 Patumwan, Bangkok 10330, Thailand

**Keywords:** biomarker, donor-specific, graft dysfunction, immune response-related, kidney-specific, kidney transplantation, urinary extracellular vesicles

## Abstract

Extracellular vesicles (EVs) are nanoparticles that transmit molecules from releasing cells to target cells. Recent studies link urinary EVs (uEV) to diverse processes such as infection and rejection after kidney transplantation. This, and the unmet need for biomarkers diagnosing kidney transplant dysfunction, has led to the current high level of interest in uEV. uEV provide non-intrusive access to local protein, DNA, and RNA analytics without invasive biopsy. To determine the added value of uEV measurements for detecting allograft dysfunction after kidney transplantation, we systematically included all related literature containing directly relevant information, with the addition of indirect evidence regarding urine or kidney injury without transplantation. According to their varying characteristics, uEV markers after transplantation could be categorized into kidney-specific, donor-specific, and immune response-related (IR-) markers. A few convincing studies have shown that kidney-specific markers (PODXL, ion cotransporters, SYT17, NGAL, and CD133) and IR-markers (CD3, multi-mRNA signatures, and viral miRNA) could diagnose rejection, BK virus-associated nephropathy, and calcineurin inhibitor nephrotoxicity after kidney transplantation. In addition, some indirect proof regarding donor-specific markers (donor-derived cell-free DNA) in urine has been demonstrated. Together, this literature review provides directions for exploring novel uEV markers’ profiling complications after kidney transplantation.

## 1. Introduction

Kidney transplantation provides better long-term survival and quality of life compared to dialysis [1]. In Europe and the United States of America, the current 5-year kidney allograft survival rate has reached 90% for living donor kidney transplant recipients (KTR), but about 50% of KTR still suffer allograft failure within 10 years of transplantation [2,3]. Kidney function is mostly assessed by serum creatinine (SCr) concentrations, estimated glomerular filtration rate (eGFR), and urine protein concentrations. However, for the early detection of kidney allograft dysfunction (including, among others, rejection, ischemia-reperfusion injury [I/R], recurrent primary kidney disease, drug-induced nephropathy, and infections), and establishing its cause, the value of these classical biomarkers is limited [4,5]. A for-cause biopsy is the current gold standard to establish the cause of kidney allograft dysfunction. However, a for-cause biopsy is costly and invasive, and this has fueled a continuing search for alternative biomarkers. Currently, non-invasive liquid biopsy and liquid biopsy-based biomarkers, e.g., cell-free DNA and extracellular vesicles (EVs), have shown an extremely high diagnostic value in various disorders, including malignancies, autoimmune diseases, organ dysfunction, and transplantation [6,7]. In this manuscript, we discuss how measurements of EVs could provide additional information about kidney function, thereby avoiding the need for an invasive biopsy [8].

**Figure 1:**
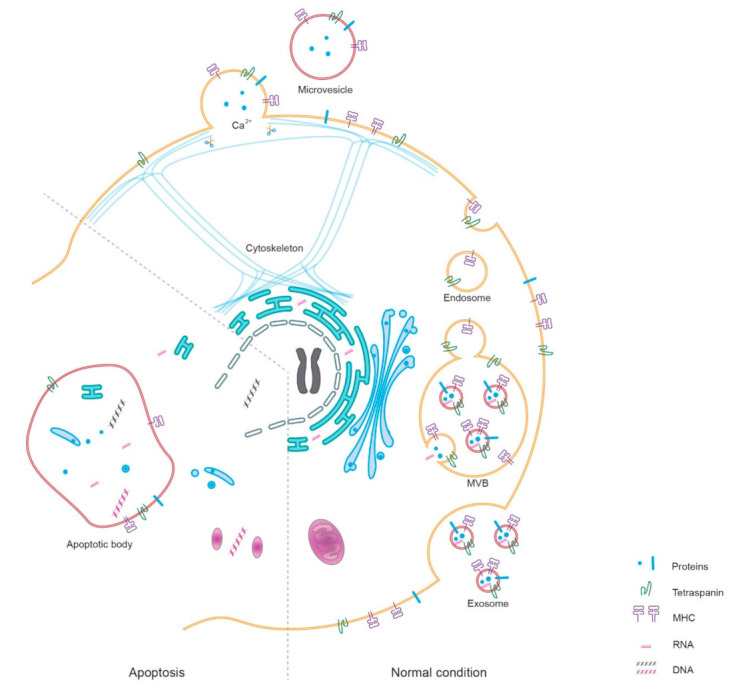
The biogenesis of extracellular vesicles (EVs). In normal conditions, exosomes are generated from the endosomal system and released by the intercellular multi-vesicle body (MVB). Microvesicles derive from the budding of plasma membrane due to the rearrangement of the cytoskeleton, with a high concentration of intracellular Ca^2+^ in the budding position. Apoptotic bodies are merely released during apoptosis. General markers such as proteins of the parental cell, tetraspanins, major histocompatibility complex (MHC), and RNA molecules can be found in all subtypes of EVs, but fragments of DNA and organelle are mostly assembled into apoptotic bodies during apoptosis.

Typical EVs have a lipid bilayer and range in diameter from 20 to 1000 nm and are generally classified into several subtypes due to different biogenesis pathways (Figure 1) [6,9]. The International Society for Extracellular Vesicles recommends the use of biochemical composition (surface markers, cargos) or parent cells to define and classify EVs [6]. EVs widely exist in various body fluids, e.g., plasma and urine, and can also be found in culture medium [6,10,11].

Urine is the most easily accessible, non-invasive body fluid for biomarker research on kidney dysfunction, including rejection. Urinary EVs (uEV) might provide highly specific information for the kidney. For example, the proteins in uEV are predominantly (99.96%) derived from the kidney and urinary tract [12]. Moreover, the changes in protein expression in kidneys are reliably assessed by the proteome of uEV [13].

Since several studies that enrolled different subjects and focused on diverse types of dysfunction have already explored the utility of uEV in kidney transplantation, our objective is to systematically summarize and investigate the diagnostic value of all types of biomarkers, such as protein, DNA, and RNA, in uEV during kidney transplantation. Due to the heterogeneity of available articles covered, with few of the same markers measured in uEV, data synthesis and meta-analysis are currently not appropriate. In spite of this, we expect this review to promote the clinical application of uEV in kidney transplantation, providing more and specific information about kidney allograft function than traditional markers (such as SCr, eGFR) and thus potentially avoiding unnecessary invasive biopsies after transplantation.

## 2. Materials and Methods

### 2.1. Data Sources and Searches

This systematic review was in accordance with the 2020 Preferred Reporting Items for Systematic Reviews and Meta-Analyses (PRISMA) guidelines [14]. The literature search was conducted in the databases of PubMed and Scopus on 15 September 2021. All retrieved articles were also manually reviewed for studies in their references that could possibly be included.

For PubMed, we used the following terms: (“exosomes” [Mesh] OR “extracellular vesicles” [Mesh] OR “cell-derived microparticles” [Mesh]) AND “kidney transplantation” [Mesh] AND (“urine” [Subheading] OR “urinary” [All]). For Scopus, the following terms were used: TITLE-ABS-KEY (urine AND ((kidney AND transplantation) OR (kidney AND transplant)) AND ((extracellular AND vesicle) OR exosome OR ectosome OR microvesicle OR (apoptotic AND body))).

### 2.2. Study Selection and Eligibility Criteria

Retrospective and prospective studies that investigated the utility of uEV in kidney transplantation were included. Studies that only used animal models or cultured cells, or no uEV samples from kidney transplantation, were excluded. Only studies that showed a potential diagnostic value of markers in uEV during human kidney transplantation were included. Two authors (L.W. and S.U.) independently screened the titles and abstracts of the electronic citations, and full-text articles were retrieved for comprehensive review and independently rescreened. Disagreements were resolved through consensus and arbitration by K.B., D.H., and C.B.

### 2.3. Data Extraction and Quality Assessment

The following data were extracted from each study: author’s name, year of publication, country of origin, outcome and numbers of KTR, timing of uEV measurement, method of uEV measurement, normalization method of urine dilution or concentration of uEV, type of marker detected in uEV, and the potential diagnostic information provided by uEV marker. Due to the high heterogeneity of included articles, quantitative synthesis of data was not appropriate. The assessment of reference was conducted with the Strengthening the Reporting of Observational Studies in Epidemiology (STROBE) statement for observational studies [15].

## 3. Results

**Figure 2:**
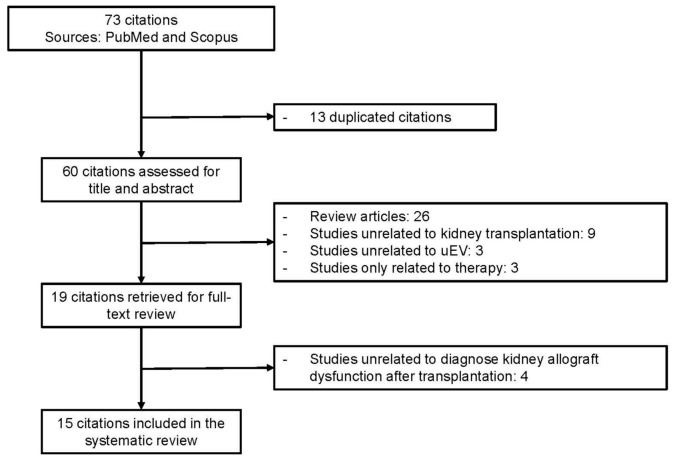
Flow diagram of the study selection.

In total, 73 published papers were retrieved. The flow diagram of the included and excluded articles is shown in Figure 2. After the exclusion of duplicated and irrelevant studies, 15 articles underwent data extraction. All included articles are shown with extracted data in Table 1. The STROBE assessment for each study is shown in the Supplement (Table S1). As shown in Table 1, 5 studies enrolled fewer than 30 KTR in total. Overall, 6 studies attempted to normalize the dilution of urine or concentration of uEV, whilst 9 studies did not mention any efforts regarding normalization.

## 4. Discussion

**Figure 3:**
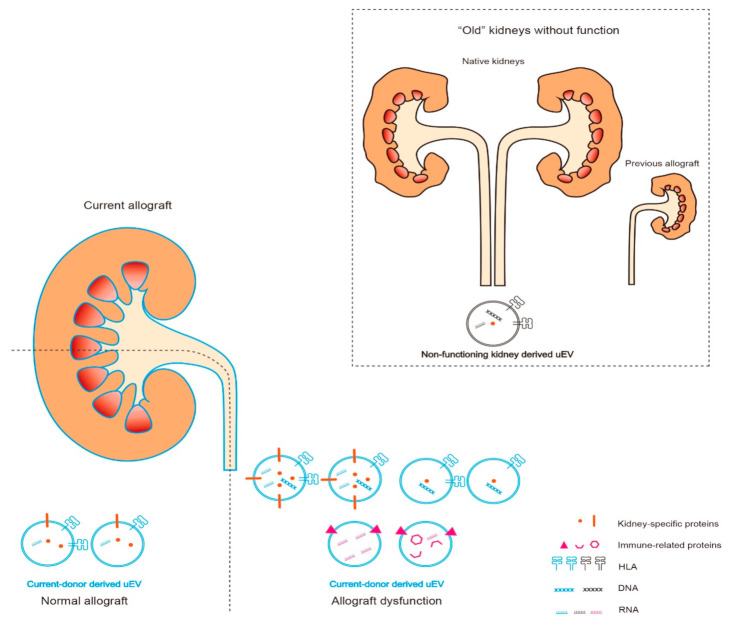
Urinary extracellular vesicles (uEV) in kidney transplantation. The “old” kidney/non-functioning kidney-derived vesicles, human leukocyte antigen (HLA), DNA, and RNA are denoted in black. Current-donor-derived vesicles, HLA, DNA, and RNA, are denoted in blue. Kidney-specific proteins are denoted in orange, and immune-related (including viral molecules) are denoted in pink. The “old” kidneys, including recipient native kidneys and previous kidney allograft, might generate fewer uEV than the currently functional kidney allograft. During allograft dysfunction, the population of uEV and the expression of markers in uEV would be different from those in normal condition.

Due to their different characteristics, we classify the diagnostic markers in uEV into three subtypes: (1) kidney-specific, (2) donor-specific, and (3) immune response-related (IR-) markers, thereby helping an understanding of their intrinsic biogenesis and properties during kidney transplantation (Figure 3). In addition, some important findings or clues that are not included in Table 1 are introduced in this review to help highlight the directions for future studies.

Kidney-specific markers are of great value in studying kidney allograft function and could reflect minimal changes, such as age-associated changes of the kidney in living donors [30,31,32]. Donor-specific markers could be identified by human leukocyte antigen (HLA) or specific gene sequences in nucleic acid cargos, according to differences in the genetic make-up between donor and recipient. Hence, donor-specific markers in uEV might have the highest specificity for a current allograft, although no direct evidence has been forthcoming as yet. Immune reactions are strongly associated with the release of uEV, which infiltrate immune cells or pathogen-containing cells in the kidney allograft, and IR- markers in uEV can provide specific information during rejection and infection [33,34,35].

### 4.1. Kidney Specific Markers

#### 4.1.1. The Biogenesis of Kidney Specific Markers

The current functioning kidney should be the major derivation of uEV because “old” kidneys, i.e., the native kidneys of the recipient, and those from previous donors, have little functionality due to barely integrated nephrons, although these might generate a small number of uEV (Figure 3).

In healthy conditions, kidney-specific proteins, e.g., aquaporin-1 (AQP1), aquaporin-2 (AQP2), Na-Cl cotransporter (NCC), Na-K-Cl cotransporter 2 (NKCC2), and PODXL, show high abundance in uEV [36,37]. During low kidney functionality, decreased release of uEV-AQP1 and uEV-AQP2 effectively detect GFR in G4 and G5 categories (KIDIGO guidelines) with a high area under the curve (AUC) value of 0.945 in the receiver operator characteristics curve analysis [38]. UEV-AQP1 has an extremely low level (not visible on Western blot) in patients with end-stage renal disease (ESRD) but shows a small increase after kidney transplantation [16,39]. Before transplantation, uEV-AQP2 is also barely detectable in KTR but increases significantly post-transplant [39]. Therefore, these kidney-specific markers within uEV might be mostly and specifically excreted by the functional kidney allograft.

#### 4.1.2. The Diagnostic Potential of Kidney Specific Markers

Compared to uEV-AQP2, the lower increase of uEV-AQP1 might be associated with early I/R [16]. Sonoda et al. set up an I/R rat model and found that decreased uEV-AQP1 occurred from 6 h to 96 h after an I/R operation compared to the sham group [16]. The decreased expression level of uEV-AQP1 was not associated with AQP1 expression in kidney tissue and the total protein in the uEV, and so might be caused by the reduced excretion of AQP1 into the uEV [16]. The authors also found that uEV-AQP1 showed no association with proteinuria in patients with nephropathy, suggesting a specific diagnostic value of uEV-AQP1 for I/R [16].

Pisitkun et al. reported a proteomic study that found elevated expression of renal cotransporters in uEV of KTR with tubular injury when compared to KTR overall [17] (Table 1). Elevated uEV-NCC and uEV-NKCC2 were discovered in KTR treated with calcineurin inhibitors (CNIs), including cyclosporine A (CsA) and tacrolimus, compared to CNI-free subjects [20,24]. In CsA recipients, uEV-NCC and uEV-NKCC2 showed strong correlations with CsA blood concentration, synchronously indicating CsA nephrotoxicity [20]. Moreover, after kidney transplantation, proteinuria had positive correlations with the glycosylated γENaC and furin-cleaved γENaC in uEV [25].

A recent uEV-proteomic study demonstrated that PODXL was one of the top 10 highly expressed proteins in small uEV of KTR with no early graft dysfunction [37]. Compared to KTR with stable allograft function, KTR with acute T cell-mediated rejection (TCMR) showed significantly decreased PODXL in uEV [26]. In KTR with chronic antibody-mediated rejection (CAMR) and severely declined kidney function (mean SCr 3.6 mg/dL), PODXL was also one of the most decreased proteins in uEV [28].

CD133 is highly expressed in progenitor-like cells of nephrons that release CD133+ uEV, indicating proliferation in healthy subjects or recovery after injury [19,40]. In the urine of ESRD patients, CD133+ uEV was hardly detectable but was significantly increased on day 7 after kidney transplantation. A smaller increase compared to KTR with early allograft function might indicate delayed graft function (DGF) due to vascular lesion [19] (Table 1).

Synaptotagmin-17 (SYT17) is also specifically and highly expressed in the kidney. After kidney transplantation, the expression level of SYT17 in urinary exosomes was specifically elevated during CAMR compared to that in normal conditions, renal tubulointerstitial lesions, or CsA nephrotoxicity [29] (Table 1). The general tetraspanin marker CD9 was used to set up a normalization of urine dilution and showed that SYT17/CD9 could diagnose CAMR with a high AUC value of 0.82.

Neutrophil gelatinase-associated lipocalin (NGAL) is a typical kidney-specific marker for kidney injury. In the urinary exosomes from KTR, the deceased donor group showed a significantly higher level of NGAL than the living donor group 3 days after transplantation [18]. KTR who developed DGF also showed much higher urinary exosomal NGAL than those with no DGF [18] (Table 1).

According to these previous findings, we speculate that the expression of kidney-specific proteins in uEV might share some common characteristics during kidney transplantation: (1) have a detectable and stable level in healthy subjects, (2) become barely detectable during ESRD due to few surviving parent cells in the “old” kidneys, (3) increase to a higher level than the ESRD period after kidney transplantation depending on the quality of allograft kidney, (4) temporarily show an increase or decrease during different types of kidney injury, (5) become barely detectable when the KTR develops ESRD again after serious or multiple kidney injury.

### 4.2. Donor Specific Markers

#### 4.2.1. HLA

A proteomics study first reported that HLA-A, -B, -DRA, -DRB3 could indeed be measured in urinary exosomes from healthy individuals [41]. Park et al. used magnetic beads to catch T cell-derived uEV and found that HLA-ABC showed abundant expression in these uEV [23]. The urinary exosome also contains other subtypes/precursors of HLA, including the HLA-B, -DMA, -DMB, -DRA, and -A1 alpha chain, that were shown to be elevated in KTR with antibody-mediated rejection (AMR), compared to those with no AMR [17]. However, the quantity of HLA subclasses in uEV might be variable [42]. For instance, in podocyte vesicle-enriched urinary samples, HLA-B41,-B54,-B59 showed higher expression levels than HLA-A3, -A24, -A29, and HLA class II molecules had lower expression levels than those of HLA class I [42]. Donor-specific uEV measurements based on HLA mismatch might be hindered by the variable expression of HLA. To arrive at good and reliable measurements, we suggest that uEV should be isolated or concentrated from the urine before uEV-HLA analysis.

#### 4.2.2. Cell-Free DNA

In addition to cell surface uEV markers, intra uEV markers are also of importance in kidney transplantation. Cell-free DNA (cf-DNA) represents a diagnostic marker for allograft dysfunction for several years, with donor-specific genetic sequences defining donor-derived cell-free (dd-cfDNA) [43,44]. For example, single nucleotide polymorphisms and InDels (genetic insertions or deletions) could be used to identify dd-cfDNA [45,46].

DNA is abundant in apoptotic bodies derived from damaged cells, possibly explained by nuclear and mitochondrial fragmentation [47,48] (Figure 1). Intracellular stress could also promote chromosomal DNA and mitochondrial DNA (mtDNA) assembling into other subtypes of EV [49]. Fernando and colleagues found that 93% plasma cf-DNA was localized in plasma exosomes [50]. Currently, there is limited research on cf-DNA or dd-cfDNA in uEV, but uEV as a derivation or localization of urinary cf-DNA in cell-free urine samples could be considered. A previous study found that, compared to KTR with normal eGFR, dd-cfDNA in urine was significantly elevated in KTR with acute and chronic rejection and BK virus-associated nephropathy (BKVN) [51].

uEV-mtDNA is an interesting subtype of cf-DNA in uEV, especially in those KTR with pre-transplant diabetes. A cohort study showed that, despite strict glycemic control, KTR with pre-transplant diabetes still showed a high incidence (47.7%) of recurrent diabetic nephropathy (DN) 5 years after transplant [52]. In urinary exosomes, mtDNA was significantly decreased in patients with DN (36 ± 18 copies/ng) in contrast to healthy subjects (432 ± 147 copies/ng) [53]. In urine, mtDNA had an inverse association with elevated eGFR during DN [54]. These findings corresponded, implying that donor-specific mtDNA might represent a potential marker in uEV for recurrent DN.

### 4.3. Immune Response-Related Markers in uEV

#### 4.3.1. Proteins

Classical protein markers for leukocytes and T cells are detectable in uEV, but we should be aware that common immune-related markers, such as CD3 and CD45, in uEV might barely distinguish rejection from other types of immune-related allograft dysfunction. CD3+ uEV show strong positive correlations with kidney allograft rejection [23]. However, in renal tissue, CD3+ EVs and CD45+ EVs are also significantly elevated in hypertension-related kidney injury [55]. Moreover, CD3+ T cells markedly infiltrate renal tubules and interstitium during DN, leading to chronic inflammation [56].

In contrast, the complement components may provide more specific information. In uEV, complement components 3 (C3), C4-a, C4-b were significantly increased in KTR with CAMR, compared with recipients with long-term graft survival [28]. C3 also showed the second highest correlation with TCMR among 63 overexpressed proteins in the uEV from KTR [26]. In addition, the overexpression of C1q subcomponent subunit B and C1r in uEV was strongly correlated with decreased GFR in the first year after kidney transplantation [37]. During CNI nephrotoxicity, a condition of immunosuppression, the levels of complement factors C3, C5, C7, C9 in uEV of KTR were significantly lower compared to KTR with interstitial fibrosis and tubular atrophy (IFTA), although only 17 KTR were enrolled [27]. These findings imply that the uEV-complement might be specifically correlated with the host immune response, especially rejection.

In uEV, defensin-alpha 5, chloride channel accessory 1, apolipoprotein A-2, CD5 molecule-like, alpha-2-macroglobulin, protein S, immunoglobulin heavy constant mu, and the heat shock protein family were all elevated in KTR with acute rejection in contrast to non-rejection patients [17,21]. Compared to KTR with stable kidney function, hemopexin and apolipoprotein A-1 in uEV were both significantly increased in KTR with CAMR [28] or acute TCMR [26]. In addition, the polymeric immunoglobulin receptor in uEV was significantly increased in TCMR but slightly decreased in CAMR, which might help distinguish CAMR from TCMR [26,28].

#### 4.3.2. RNA

uEV represent a major localization of cell-free RNA in the urine due to the membrane protection from RNase [56,57]. In 2013, Crescitelli et al. first demonstrated RNA profiles in subtypes of EV [58]. They found that RNA was primarily assembled into exosomes and apoptotic bodies, not into microvesicles, and cell injury could promote this assembly (Figure 1) [58]. uEV immune-related messenger RNA (mRNA) showed a strong association with kidney allograft dysfunction, especially rejection and viral infection [8]. Micro RNA (miRNA)—that has clear correlations with the immune response—could also be found in uEV [59].

Recently, Fekih et al. reported that a multi-mRNA signature in uEV could discriminate 59 KTR with biopsy-proven acute rejection from those without rejection [8]. In uEV, a signature consisting of 15 different mRNA could diagnose rejection with a high AUC value of 0.93. In addition, the mRNA signature of C3-CD44-CD74-CD119-CXCL11 was significantly higher in AMR compared to TCMR [8]. As for chronic inflammation, the elevated CCL21-mRNA in uEV might be a candidate biomarker for early recurrent diabetic nephropathy based on a recent study. In uEV, CCL21-mRNA showed a high AUC value of 0.89 for distinguishing diabetic patients with early biopsy-proven DN (eGFR > 90 mL/min/1.73 m^2^) from those without DN [56]. These are novel findings in the uEV field and should be further confirmed in future studies with more KTR samples.

miRNA is clearly associated with the immune response during kidney transplantation [60], and the miRNome of uEV has been well demonstrated before transplantation. In 2013, Gildea and team first reported a miRNome of urinary exosome where less than 21 serum-exosome-derived miRNA were detected in a total measured 1898 miRNA, suggesting that uEV-miRNA might derive mostly from the kidney or urinary tract [61]. In 2018, another miRNome study of uEV from donors reported that the profile of miRNA did not discriminate between living donors and deceased donors before transplantation [62]. The role of miRNA in uEV after kidney transplantation is not yet fully explored as currently most diagnostic findings of miRNA are based on human kidney disease before transplantation [59], cultured kidney cell [30,63], animal model [63,64], or urine sample only with no uEV isolation or detection [57,65,66,67,68], rather than on uEV from KTR after transplantation.

For example, uEV-miR-200 and uEV-miR-29 were positively correlated with eGFR and renal fibrosis during chronic kidney disease [59], which should be noticed in KTR with chronic kidney injury. During chronic injury, the damaged tubular epithelia released increased miR-21-enriched uEV that further exacerbate renal fibrosis [63], suggesting that uEV-miRNA might also be a crucial modulator in the damaged kidney allograft.

Some studies focused on the miRNA in the urine and not on uEV. However, uEV-miRNA might be a major localization/derivation of total urinary miRNA [57], where urinary miR-21 is especially highlighted during kidney transplantation. Compared to a group with normal function, KTR with IFTA showed upregulated urinary miR-21 [65], which corresponded to elevated uEV-miR-21 during chronic kidney disease [63]. Khalid et al. reported that elevated urinary miR-21 could predict DGF with an AUC between 0.75 to 0.94 [66]. In the first urine from KTR after transplantation, miR-21 was elevated at least 5-fold in deceased donors with DGF in contrast to deceased donors with no DGF (*p*-value > 0.001 and <0.05), or living donors with no DGF (*p*-value < 0.001) [66]. In addition, deregulated urinary miR-142-3p and miR-200b were found in KTR with IFTA [67]. Urinary miR-210 was strongly deregulated in KTR, with acute TCMR predicting a decline in GFR in the first year after transplantation [68].

#### 4.3.3. Viral Infection Related Markers

Viral components can be assembled into the uEV after transplantation when the kidney allograft is infected by a virus hampering kidney function (Figure 3). BK virus nephropathy is a common outcome of viral infection after kidney transplantation [69]. In uEV, Kim et al. found that elevated viral miR-B1-5p, miR-B1-3p, and recipient miR-16 could precisely diagnose BKVN, and the uEV-miR-B1-5p showed a high AUC value of 0.989 [22]. Their findings partially corresponded with another study, where miR-B1-5p and miR-B1-3p were also significantly increased in KTR with BKVN, in contrast to those with no BKVN. However, kidney biopsy tissues, not uEV, were used in this study [70].

Coronavirus disease 2019 (COVID-19) related nephropathy is another recent important viral nephropathy during kidney transplantation [71]. Due to immunosuppression, KTR might more likely develop severe COVID-19 than the general population [71,72]. In about a third of patients with COVID-19, the severe acute respiratory syndrome coronavirus 2 (SARS-CoV-2) could infect kidney tissue and lead to acute kidney injury [73]. SARS-CoV-2 is hardly detected in the urine [74], but SARS-CoV-2 infected kidney cells might release more uEV with specific markers. In vitro, the spike protein of SARS-CoV-2 could promote embryonic kidney cells (HEK 293) to release EVs with higher expression of miR-590-3p and miR-148a [75].

### 4.4. Challenges and Future Perspective

uEV have potential as non-invasive biomarkers to monitor kidney allograft dysfunction and to establish its cause. However, there are still two major blanks to be filled, with potential for new and interesting directions of study: (1) HLA and DNA have not yet been shown to be “donor-specific” in uEV, based on any evidence of donor-recipient mismatch; (2) urinary miRNA is of great diagnostic value for kidney allograft dysfunction, whereas the characteristics of uEV-miRNA in kidney transplantation are unknown. In addition, tacrolimus is currently more widely used than CsA, so future CNI-related research should also focus on tacrolimus-related complications.

At present, the difficulty with uEV studies is that, although available research explores interesting diagnostic markers in uEV, these studies are not all of the same quality. Challenges relating to this:

1. many studies omit the necessity of normalizing urine dilution (Table 1);
2. some studies merely detect the expression level of markers in the total uEV, rather than measure a particular “marker”-positive population of uEV;
3. some clinical research is limited by small numbers of samples or a poor or non-existent description regarding the clinical properties of enrolled patients;
4. the significant proteomic changes of uEV in some types of kidney dysfunction have been well studied, whereas the diagnostic accuracy (sensitivity, specificity, and AUC) of these changes is still unclear.

We propose the following steps to move uEV research forward:

1. The normalization of uEV concentration/urine dilution is necessary in the research protocol. According to the newest guidelines published by the Urine Task Force of the International Society for Extracellular Vesicles, variable dilution of urine is a major challenge in uEV research [76]. Whether in healthy or unwell subjects, the concentration of uEV is highly influenced by water-loading [77]. For this, two methods are commonly recommended to realize normalization: (1) the relative excretion rate is based on the ratio between target-marker and other markers of uEV (e.g., numbers, the total yield of protein or RNA, tetraspanin, prostate-specific markers, etc.); (2) the absolute excretion rate is based on a long-term collection of urine or the ratio between target-marker and urinary creatinine [76].
2. The measurement of markers in uEV mostly depends on isolation. This could cause bias, especially loss or selection of uEV due to diverse methods, e.g., ultracentrifugation, size exclusion chromatography, precipitation [78,79,80]. More efforts are still needed to compare the yield and purity of uEV between techniques. To avoid isolation bias, more sensitive techniques, such as imaging flow cytometry, might provide the possibility of independence of isolation, also facilitating the detection of a particular population of uEV [81], especially the donor-specific HLA-positive uEV [78]. Analysis of subpopulations of uEVs will reveal their specific contribution in clinical events by zooming in on uEV subpopulations; their role in rejection and other complications after transplantation will then become evident. This improves the value of uEV measurements like Western blot and immunoblot analysis.
3. The data is based on small, single-center studies, which makes it difficult to draw conclusions. Future studies should take a multi-center approach which enables large patient numbers. This should take into account clinical properties including (1) gender, race, age, body size, HLA-mismatch, and common chronic diseases (e.g., hypertension, diabetes mellitus, and hepatitis virus infection) of donor and recipient; (2) the type and proportion of donors, e.g., living donor or deceased donor; (3) immunosuppressor-treated or -free, and the type of medicine administered. This information is of critical value for multivariate analysis, which will provide results defining the diagnostic accuracy of uEV measurement in kidney transplantation.

## 5. Conclusions

This review highlights potential uEV-marker candidates and indicates directions for further study, opening up an attractive perspective that would universally utilize uEV in clinical practice and avoid unnecessary biopsy after kidney transplantation. Both uEV surface markers and cargos provide an abundance of information regarding the health of renal parenchymal cells and the immune response towards the kidney allograft. As a representative type of liquid biopsy, uEV might also be widely used in other diseases in the future.

## Figures and Tables

**Table 1:** Summary of studies reporting data of the diagnostic value of uEV in kidney transplantation.

Authors	Year of Publication	Country of Origin	Outcomes and Population	Timing of uEV Measurement	Technique	Normalization	Marker in uEV	Diagnostic Information
Sonoda et al. [16]	2009	Japan	Early I/R after transplantation (*n* = 1)	Day 1 before and day 2, 6 after transplantation	Immunoblot analysis	No	AQP1	AQP1 was hardly detectable before transplantation and significantly increased on day 2 after transplantation; Decreased AQP1 on day 6 was likely caused by I/R, according to findings in animal I/R models.
Pisitkun et al. [17]	2012	USA	Nonspecific findings (*n* = 2); TI (*n* = 7); TCMR (*n* = 6); AMR (*n* = 3)	Biopsy after transplantation	Large-scale liquid chromatography-tandem mass spectrometry	No	Proteome	Compared to all KTR: Cotransporter family showed higher expression in TI; Proteins related to epithelial cell differentiation showed higher expression in TCMR; Proteins related to acute inflammatory response, or antigen processing and presentation showed higher expression in AMR.
Alvarez et al. [18]	2013	Chile	Non-DGF (*n* = 12); DGF (*n* = 3)	Day 1, 2, 3 after transplantation	Western blotting	No	NGAL	Deceased donors showed higher levels of NGAL than living donors; The DGF group showed higher levels of NGAL than the non-DGF group.
Dimuccio et al. [19]	2014	Italy	Early graft function (*n* = 13); DGF (*n* = 12)	Hour 6, day 1, 7, 30 after transplantation	Cytofluorimetric analysis & Western blotting	Ratio of the expression level in CD133+ uEV and in total uEV	CD133	The proportion of CD133+ uEVs was much higher in healthy controls than ESRD patients; CD133+ uEVs increased from day 1 to day 30 after transplantation; KTR with DGF showed a lower increase in CD133+ uEV at day 1; The lower level of CD133+ uEV were associated with worse graft vascular lesions.
Esteva-Font et al. [20]	2014	Spain	CsA-free (*n* = 8); CsA-treated (*n* = 39)	1 year after transplantation	Immunoblotting	24-h urine volume	NCC & NKCC2	NCC and NKCC2 was more highly expressed in the CsA-treated group, whereas the *p*-values of all were above 0.05 (NCC: 0.1315, NKCC2 0.0542); NCC and NKCC2 were positively associated with the blood concentration of CsA, with *p* values of 0.0152 and 0.0497, respectively.
Sigdel et al. [21]	2015	USA	No rejection (*n* = 20); acute rejection (*n* = 10)	Biopsy after transplantation	Isobaric tags for relative and absolute quantitation & nanoLC-MS/MS	No	Proteome	DEFA5, CD5L, APOM, A2M, APOA2, PROS1, IGHM, FGA, and FGB were significantly increased in the acute rejection group.
Kim et al. [22]	2017	South Korea	Normal (*n* = 15); BKVN (*n* = 13); TCMR (*n* = 27); acute AMR (*n* = 9); CAMR (*n* = 16)	Biopsy after transplantation	Quantitative real-time polymerase chain reaction	MiR-16	Viral microRNA	Increased bkv-miR-B1-5p could diagnose BKVN, with AUC value of 0.989 and cut-off value of 5.9 log10 copies/mL (sensitivity 100%; specificity 98.5%); Increased bkv-miR-B1-5p/miR-16 could diagnose BKVN, with AUC value of 0.985 and cut-off value of 1.2 log10 copies/mL (sensitivity 100%; specificity 98.5%).
Park et al. [23]	2017	USA	No rejection (*n* = 22); acute rejection (*n* = 22)	Biopsy after transplantation	Integrated kidney exosome analysis	No	CD3	Increased CD3 could diagnose acute rejection with AUC value of 0.911 and cut-off value of 0.298 μA (sensitivity 92.8%, specificity 87.5%) in discovery set, with AUC value of 0.837. (sensitivity 63.6%, specificity 100%) in validation set.
Tutakhel et al. [24]	2017	Netherlands	CNIs-free (*n* = 13); CsA-treated (*n* = 9); Tacrolimus-treated (*n* = 23)	At least 6 months after transplantation	Immunoblotting	Ratio of phosphorylated NCC and total NCC	NCC	The expression of total NCC or phosphorylated NCC (Thr60) in CNI-treated KTR was significantly higher.
Hinrichs et al. [25]	2018	Denmark	No albuminuria (*n* = 19); albuminuria (*n* = 18)	1 year after transplantation	Western blotting	Urinary creatinine	γENaC	The expression of furin-cleaved γENaC and protease-cleaved γENaC (not full-length γENaC) was significantly increased in KTR with albuminuria.
Lim et al. [26]	2018	South Korea	Normal (*n* = 22); TCMR (*n* = 25)	Biopsy after transplantation	nanoLC-MS/MS & Western blotting	No	Proteome	APOA1, complement C3, HPX, PIGR, RBP4, etc. were increased in acute TCMR; NEP, PROM1, LRP2, CD9, NAPSA, etc., were decreased in TCMR.
Carreras-Planella et al. [27]	2020	Spain	Normal (*n* = 7); CNIs nephrotoxicity (*n* = 5); IFTA (*n* = 5)	Biopsy after transplantation	Mass spectrometry	Ezrin	Proteome	Compared to IFTA: Uroplakin family (UPK1A, UPK1B, UPK2, UPK3A), RAB1B, etc. were more positive in CNIs nephrotoxity; Complement components (C3, C5, C7) etc. were more negative in CNIs nephrotoxity.
Jung et al. [28]	2020	South Korea	Long-term graft survival (*n* = 57); CAMR (*n* = 26)	Biopsy after transplantation	Liquid chromatography–mass spectrometry	No	Proteome	PODXL, MUC1, etc. were decreased in CAMR; TTR, APOA1, HPX, complement C3, C4a, C4b, etc., were increased in CAMR.
Takada et al. [29]	2020	Japan	Normal (*n* = 20); IFTA (*n* = 19); CNIs nephrotoxicity (*n* = 17); CAMR (*n* = 22)	Biopsy after transplantation	Western blotting	CD9	SYT17	The ratio of SYT17/CD9 could distinguish CAMR from other groups, with AUC value of 0.82 and cut-off value of 0.42 (sensitivity 77%; specificity 87%)
Fekih et al. [8]	2021	USA	No rejection (*n* = 133); acute AMR (*n* = 8); CAMR (*n* = 16); TCMR (*n* = 35); borderline TCMR (*n* = 23); BKVN (*n* = 5)	Biopsy after transplantation	Quantitative real-time polymerase chain reaction	No	Messenger RNA	15 mRNA (CXCL11, CD74, IL32, etc.) could discriminate rejection from non-rejection, with the AUC value of 0.93 (sensitivity 84.7%; specificity 94%); 5 mRNA (CD74, complement C3, CXCL11, CD44, and IFNAR2) could distinguish TCMR from ABMR, with AUC value of 0.87 (sensitivity 87.5%; specificity 82.9%).

I/R: ischemia-reperfusion injury; AQP1: aquaporin 1; TCMR: T cell-mediated rejection; AMR: antibody-mediated rejection; TI: tubular injury; KTR: kidney transplant recipients; HLA: human leukocyte antigen; DGF: delayed graft function; NGAL: neutrophil gelatinase-associated lipocalin; CsA: cyclosporine A; NCC: sodium-chloride cotransporter; NKCC2: Na-K-Cl cotransporter; nanoLC-MS/MS: nano-scale liquid chromatography-tandem mass spectrometry; DEFA5: defensin-alpha 5; CD5L: CD5 molecule-like; APOM: apolipoprotein M; A2M: alpha-2-macroglobulin; APOA2: apolipoprotein A2; PROS1: protein S; IGHM: immunoglobulin heavy constant mu; FGA: fibrinogen alpha chain; FGB: fibrinogen beta chain; BKVN: BK virus associated nephropathy; CAMR: chronic antibody-mediated rejection; AUC: area under the curve; CNIs: calcineurin inhibitors; ENaC: epithelial sodium channel; APOA1: apolipoprotein A1; HPX: hemopexin; PIGR: polymeric immunoglobulin receptor; RBP4: retinol binding protein 4; NEP: neprilysin; PROM1: prominin 1; LRP2: low density lipoprotein receptor-related protein 2; NAPSA: napsin A aspartic peptidase; IFTA: interstitial fibrosis and tubular atrophy; SYT17; synaptotagmin 17; PODXL: podocalyxin; MUC1: mucin 1; TTR: transthyretin; RAB1B: Ras-related protein rab-1B; CXCL11: C-X-C motif chemokine ligand 11; IL32: interleukin 32; IFNAR2: interferon alpha and beta receptor subunit 2.

## Data Availability

Not applicable.

## Supplementary Materials

The following are available online at https://www.mdpi.com/article/10.3390/ijms221910499/s1, Table S1: Assessment of reference according to the STROBE statement.
