# Supplementary material for: Urinary Extracellular Vesicles Are a Novel Tool to Monitor Allograft Function in Kidney Transplantation: A Systematic Review
Source: Int J Mol Sci. 2021 Sep 28;22(19):10499. doi: 10.3390/ijms221910499 (PMC8508981; doi:10.3390/ijms221910499)
Supplement: Supplementary file 1 [file ijms-22-10499-s001.zip › ijms-1399475-supplementary.pdf]

Table S1. Assessment of reference according to the STROBE statement.

		Introduction		Methods									Results					Discussion				Other
**Reference**	**1.**	**2.**	**3.**	**4.**	**5.**	**6.**	**7.**	**8.**	**9.**	**10.**	**11.**	**12.**	**13.**	**14.**	**15.**	**16.**	**17.**	**18.**	**19.**	**20.**	**21.**	**22.**
Sonoda et al. [1]	✓	✓	✓	✓	✓	✗	✗	✓	✗	✓	✗	✗	✗	✗	✗	✗	✗	✓	✓	✗	✓	✗
Pisitkun et al. [2]	✓	✓	✓	✓	✓	✓	✓	✓	✓	✓	✓	✓	✓	✗	✓	✓	✗	✓	✗	✓	✓	✓
Alvarez et al. [3]	✓	✓	✓	✓	✓	✓	✓	✓	✓	✓	✓	✗	✓	✓	✓	✓	✓	✓	✓	✓	✓	✓
Dimuccio et al. [4]	✓	✓	✓	✓	✓	✓	✓	✓	✓	✓	✓	✓	✓	✓	✓	✓	✓	✓	✓	✓	✓	✓
Esteva-Font et al. [5]	✓	✓	✓	✓	✓	✓	✓	✓	✓	✓	✓	✓	✓	✓	✓	✓	✓	✓	✓	✓	✓	✓
Sigdel et al. [6]	✓	✓	✓	✓	✓	✓	✓	✓	✓	✓	✓	✓	✓	✓	✓	✓	✓	✓	✓	✓	✓	✓
Kim et al. [7]	✓	✓	✓	✓	✓	✓	✓	✓	✗	✓	✓	✓	✓	✓	✓	✓	✓	✓	✗	✓	✓	✓
Park et al. [8]	✓	✓	✓	✓	✓	✓	✓	✓	✗	✓	✓	✓	✓	✓	✓	✓	✓	✓	✓	✓	✓	✓
Tutakhel et al. [9]	✓	✓	✓	✓	✓	✓	✓	✓	✓	✓	✓	✓	✓	✓	✓	✓	✓	✓	✓	✓	✓	✓
Hinrichs et al. [10]	✓	✓	✓	✓	✓	✓	✓	✓	✓	✓	✓	✓	✓	✓	✓	✓	✓	✓	✓	✓	✓	✓
Lim et al. [11]	✓	✗	✓	✓	✓	✓	✓	✓	✗	✓	✓	✓	✓	✓	✓	✓	✓	✓	✓	✓	✓	✓
Carreras-Planella et al. [12]	✓	✓	✓	✓	✓	✓	✓	✓	✓	✓	✓	✓	✓	✓	✓	✓	✓	✓	✓	✓	✓	✓
Jung et al. [13]	✓	✓	✓	✓	✓	✓	✓	✓	✗	✓	✓	✓	✓	✓	✓	✓	✓	✓	✓	✓	✓	✓
Takada et al. [14]	✓	✓	✓	✓	✓	✓	✓	✓	✗	✓	✓	✓	✓	✓	✓	✓	✓	✓	✗	✓	✓	✓
Fekih et al. [15]	✓	✓	✓	✓	✓	✓	✓	✓	✓	✓	✓	✓	✓	✓	✓	✓	✓	✓	✓	✓	✓	✓

Red cross (✗): not meeting the recommendation; Black tick (✓): meeting the recommendation.

References

1. Sonoda, H.; Yokota-Ikeda, N.; Oshikawa, S.; Kanno, Y.; Yoshinaga, K.; Uchida, K.; Ueda, Y.; Kimiya, K.; Uezono, S.; Ueda, A.; et al. Decreased Abundance of Urinary Exosomal Aquaporin-1 in Renal Ischemia-Reperfusion Injury. *Am. J. Physiol. - Ren. Physiol.* **2009**, *297*, F1006–F1016.

2. Pisitkun, T.; Gandolfo, M.T.; Das, S.; Knepper, M.A.; Bagnasco, S.M. Application of Systems Biology Principles to Protein Biomarker Discovery: Urinary Exosomal Proteome in Renal Transplantation. *Proteomics - Clin. Appl.* **2012**, *6*, 268–278.

3. Alvarez, S.; Suazo, C.; Boltansky, A.; Ursu, M.; Carvajal, D.; Innocenti, G.; Vukusich, A.; Hurtado, M.; Villanueva, S.; Carreño, J.E.; et al. Urinary Exosomes as a Source of Kidney Dysfunction Biomarker in Renal Transplantation. *Transplant. Proc.* **2013**, *45*, 3719–3723.

4. Dimuccio, V.; Ranghino, A.; Barbato, L.P.; Fop, F.; Biancone, L.; Camussi, G.; Bussolati, B. Urinary CD133+ Extracellular Vesicles Are Decreased in Kidney Transplanted Patients with Slow Graft Function and Vascular Damage. *PLoS One* **2014**, *9*, e104490.

5. Esteva-Font, C.; Guillén-Gómez, E.; Diaz, J.M.; Guirado, L.; Facundo, C.; Ars, E.; Ballarin, J.A.; Fernández-Llama, P. Renal Sodium Transporters Are Increased in Urinary Exosomes of Cyclosporine-Treated Kidney Transplant Patients. *Am. J. Nephrol.* **2014**, *39*, 528–535.

6. Sigdel, T.K.; Ng, Y.W.; Lee, S.; Nicora, C.D.; Qian, W.J.; Smith, R.D.; Camp, D.G.; Sarwal, M.M. Perturbations in the Urinary Exosome in Transplant Rejection. *Front. Med.* **2015**, *2*, 57.

7. Kim, M.H.; Lee, Y.H.; Seo, J.W.; Moon, H.; Kim, J.S.; Kim, Y.G.Y.H.; Jeong, K.H.; Moon, J.Y.; Lee, T.W.; Ihm, C.G.; et al. Urinary Exosomal Viral MicroRNA as a Marker of BK Virus Nephropathy in Kidney Transplant Recipients. *PLoS One* **2017**, *12*, e0190068.

8. Park, J.; Lin, H.-Y.Y.; Assaker, J.P.; Jeong, S.; Huang, C.-H.H.; Kurdi, A.; Lee, K.; Fraser, K.; Min, C.; Eskandari, S.; et al. Integrated Kidney Exosome Analysis for the Detection of Kidney Transplant Rejection. *ACS Nano* **2017**, *11*, 11041–11046.

9. Tutakhel, O.A.Z.; Moes, A.D.; Valdez-Flores, M.A.; Kortenoeven, M.L.A.; Vrie, M. V.D.; Jelen, S.; Fenton, R.A.; Zietse, R.; Hoenderop, J.G.J.; Hoorn, E.J.; et al. NaCl Cotransporter Abundance in Urinary Vesicles Is Increased by Calcineurin Inhibitors and Predicts Thiazide Sensitivity. *PLoS One* **2017**, *12*, e0176220.

10. Hinrichs, G.R.; Michelsen, J.S.; Zachar, R.; Friis, U.G.; Svenningsen, P.; Birn, H.; Bistrup, C.; Jensen, B.L. Albuminuria in Kidney Transplant Recipients Is Associated with Increased Urinary Serine Proteases and Activation of the Epithelial Sodium Channel. *Am. J. Physiol. - Ren. Physiol.* **2018**, *315*, F151–F160.

11. Lim, J.H.; Lee, C.H.; Kim, K.Y.; Jung, H.Y.; Choi, J.Y.; Cho, J.H.; Park, S.H.; Kim, Y.L.; Baek, M.C.; Park, J.B.; et al. Novel Urinary Exosomal Biomarkers of Acute T Cell-Mediated Rejection in Kidney Transplant Recipients: A Cross-Sectional Study. *PLoS One* **2018**, *13*, e0204204.

12. Carreras-Planella, L.; Juega, J.; Taco, O.; Cañas, L.; Franquesa, M.; Lauzurica, R.; Borràs, F.E. Proteomic Characterization of Urinary Extracellular Vesicles from Kidney-Transplanted Patients Treated with Calcineurin Inhibitors. *Int. J. Mol. Sci.* **2020**, *21*, 7569.

13. Jung, H.Y.; Lee, C.H.; Choi, J.Y.; Cho, J.H.; Park, S.H.; Kim, Y.L.; Moon, P.G.; Baek, M.C.; Berm Park, J.; Hoon Kim, Y.; et al. Potential Urinary Extracellular Vesicle Protein Biomarkers of Chronic Active Antibody-Mediated Rejection in Kidney Transplant Recipients. *J. Chromatogr. B Anal. Technol. Biomed. Life Sci.* **2020**, *1138*, 121958.

14. Takada, Y.; Kamimura, D.; Jiang, J.J.; Higuchi, H.; Iwami, D.; Hotta, K.; Tanaka, Y.; Ota, M.; Higuchi, M.; Nishio, S.; et al. Increased Urinary Exosomal SYT17 Levels in Chronic Active Antibody-Mediated Rejection after Kidney Transplantation via the IL-6 Amplifier. *Int. Immunol.* **2020**, *32*, 653–662.

15. Fekih, R. El; Hurley, J.; Tadigotla, V.; Alghamdi, A.; Srivastava, A.; Coticchia, C.; Choi, J.; Allos, H.; Yatim, K.; Alhaddad, A.; et al. Discovery and Validation of a Urinary Exosome MRNA Signature for the Diagnosis of Human Kidney Transplant Rejection. *J. Am. Soc. Nephrol.* **2021**, *32*, 994–1004.
